# The Place of the Medical Officer of Health

**Published:** 1920-11-20

**Authors:** 


### MEDICAL SERVICES IN COUNTY AREAS.

### The Place of the Medical Officer of Health.

The committee in charge of the extension of medical services in Gloucestershire is now able to report definite progress in the fulfilment of the programme that was drafted some months ago. Matters have advanced far enough for treatment to be begun before the end of the present year. The provisional arrangements include the opening of out-stations in connection with nine cottage hospitals, at Almondsbury, Berkeley, Bourton-on-the-Water, Cirencester, Fairford, Hambrook, Lydney, Tetbury, and Tewkesbury. In the latter place, for some reason, the cottage hospital has not seen its way so far to co-operate in the scheme. It is also hoped to open out-stations at nine other centres in widely different areas, so that in this preliminary respect the county is fairly well organised. In regard to the capital expenditure involved, one-half has been available from a grant of £7,000 from the Red Cross Society. It is hoped to make use of the resources of Bristol University in connection with the scheme; and the committee recommends that the bacteriological and pathological work which can properly be undertaken by the big general hospitals shall be performed in co-operation with the Public Health Laboratory at Bristol University. The cost of this part of the work is not to exceed the sum of £750.

The committee has co-opted Dr. J. Middleton Martin, medical officer of health for the county. This is as it should be, since Dr. Martin has done much to support co-ordination, and indeed drafted a detailed scheme, which was described in The Hospital at the time of its appearance. In regard to the staff, the committee recommends that where there is only one doctor practising in a neighbourhood he shall act as medical officer. Where there are two or more, it is proposed that each shall act for a period of six months. These appointments are to be subject to the recommendation of the medical advisory committee.

Gloucestershire seems to be ahead of most of the counties in putting the scheme for medical services in county areas into practice. Doubtless it is reaping the reward of early preparation, and since the medical officer of health for the county was to the fore in this matter and perhaps would have attempted such extension as was possible, independently of the present more comprehensive scheme, the value of keen men in the posts of medical officers of health is abundantly apparent. We incline to think that these posts will become increasingly important to the work now started, and that round them must be grouped much of the co-ordination now desired. Hitherto they have been confined to public health work as interpreted in certain Acts of Parliament. Now that co-ordination is the order of the day, the meaning of public health work, and the duties of medical officers of health in regard thereto, are likely to develop considerably. An administrative office in the capital of the county has hitherto sufficed, and the medical officer of health has been largely confined to what may be termed urban problems. Preventive medicine, however, has come to cover much more than sanitation, or laboratory experiments. It has passed beyond the stage of concern with the water supply, epidemics, market inspection, and legislative precautions on behalf of public health. Prevention now includes not only precautionary care, but early treatment and a ready access to institutions. To secure these it is more important to avoid waste in our present system than to attempt a new one. Among all the agencies at work, the medical officer of health is a convenient centre. and if the most is made of his advantages co-ordination can develop on existing medical services to a degree that would have seemed surprising a few years ago. Besides this, medical officers of health usually escape red tape and officialism. They were trained in the voluntary hospitals, and that training is an active influence. Probably no State officials are so little tainted with officialism as our senior medical officers of health.

